# Value to the Public of State Medical Societies*Presidential address delivered before the Medical Society of Virginia, Hot Springs, September 1, 1897.

**Published:** 1897-10

**Authors:** George Ben. Johnston

**Affiliations:** Professor of Gynecology and Abdominal Surgery, Medical College of Virginia; President of the Southern Surgical and Gynecological Association


### VALUE TO THE PUBLIC OF STATE MEDICAL SOCIETIES.*

BY GEORGE BEN. JOHNSTON, M. D., LL. D.

Professor of Gynecology and Abdominal Surgery, Medical College of Virginia; President of the Southern Surgical and Gynecological Association.

I am sensible of the high honor conferred on me by my friends of the Medical Society of Virginia in elevating me to the exalted office of president, and I wish to thank them sincerely for this mark of confidence and regard.

This office imposes the duty of speaking to a mixed audience, a task not easily accomplished, for it is difficult to choose a subject alike interesting and profitable to lay and medical hearers.

Because the real reason for the existence of such organizations as this is not well understood and their importance not properly appreciated, I will dwell briefly on the "Value to the public of State Medical Societies."

There is a disposition to regard such societies as either trades unions or social organizations where scientific work is only an incident. Let me correct this fallacy at once. Our societies have no commercial aspect, and the social feature of our conventions is only secondary to the serious affairs we meet to discuss. We are organized solely to accomplish three purposes: to advance science, promote philanthropy and foster good-fellowship. The last of these propositions needs no demonstration; it is self-evident after what lookers-on have seen and will see during this meeting. The first and second require proof.

To-day there is neither a State nor a Territory in this great Union which is without its State or Territorial medical society. All are modeled on the same general plan and have the same objects in view. They vary in age from two years (Utah, founded in 1895) to one hundred and thirty-one years (New Jersey, founded in 1766). In membership they range from forty (New Mexico) to three thousand (Pennsylvania).

From replies to inquiries addressed to officers of these societies I am able to give the following trustworthy statistics:

---

*Presidential address delivered before the Medical Society of Virginia, Hot Springs, September 1, 1897.

STATE MEDICAL SOCIETIES.

| STATE. | Membership. | Med. Law Secured by State Society. | Brd. Health Secured by State Society. | Transactions, How Published. |
|---|---|---|---|---|
| Alabama | 1,200 | Yes | Yes | Book |
| Arkansas | 270 | ........... | ........... | Not published |
| California | 300 | Yes | Yes | Book |
| Colorado | 325 | Yes | ........... | Book |
| Connecticut | 623 | Yes | Yes | Book |
| Delaware | 156 | Yes | Yes | Book |
| District of Columbia | 300 | Yes | ........... | Book |
| Florida | 130 | Yes | Yes | Book |
| Georgia | 500 | Yes | ........... | Book |
| Idaho | 55 | Yes | ........... | Not published |
| Illinois | 700 | Yes | Yes | Book |
| Indian Territory | 84 | Yes | Yes | Not published |
| Iowa | 700 Aux 1700 | Yes | Yes | Book |
| Indiana | 1,800 | Yes | Yes | Book |
| Kansas | 300 | Yes | Yes | Book |
| Kentucky | 515 | Yes | Yes | Book |
| Louisiana | 500 | Yes | ........... | Book |
| Maine | 410 | ........... | Yes | Book |
| Maryland | 500 | Yes | ........... | Book |
| Massachusetts | 2,200 | Yes | Yes | Book |
| Minnesota | 500 | Yes | Yes | Journal |
| Michigan | 550 | Yes | Yes | Book |
| Mississippi | 400 | Yes | Yes | Journal |
| Missouri | 350 | Yes | Yes | Book |
| Montana | 230 | Yes | ........... | Journal |
| Nebraska | 299 | Imperfect | Imperfect | Book |
| New Hampshire | 375 | Yes | Yes | Book |
| New York | { 443*<br>880† | Yes | Yes | Book<br>Book |
| New Mexico | 40 | Yes | Yes | Not published |
| New Jersey | 821 | Yes | ........... | Book |
| North Carolina | 500 | Yes | Yes | Book |
| Ohio | 950 | Yes | Yes | Book |
| Oregon | 127 | Yes | ........... | Book |
| Pennsylvania | 3,000 | Yes | Yes | Journal |
| Rhode Island | 260 | Yes | Yes | Book |
| South Carolina | 300 | Yes | Yes | Book |
| South Dakota | 100 | Yes | Yes | Book |
| Tennessee | 400 | Yes | ........... | Book |
| Texas | 356 | Imperfect | ........... | Book |
| Utah | 81 | Improvem't | ........... | Book |
| Vermont | 235 | Yes | Yes | Book |
| Virginia | 900 | Yes | Yes | Book |
| Washington | 185 | Yes | Yes | Book |
| West Virginia | 300 | Yes | ........... | Book |
| Wisconsin | 500 | Yes | Yes | Book |

*Medical Society State of New York. †New York State Medical Association.

It will be observed that these societies have an aggregate membership of 24,570, which almost equals the numerical strength of the nation's standing army.

To the thoughtful person it must be apparent that membership in one of these societies is a valuable possession and a mark of distinction. Most rigid and exacting requirements for entrance are imposed. Creed, political affinities and social status are ignored. The gauge is laid on merit. Sound morals, good citizenship, professional rectitude and technical skill are demanded. This standard of moral and intellectual excellence brings into their ranks only the best element of the profession; men whose character and attainments entitle them to rank with the best in their communities, whose professional acumen and honesty fit them to discharge the duties of their high calling with confidence, prudence and skill, and whose trained faculties constitute them the guardians of the public health.

I can not help believing that if our aims and purposes were better understood by the public our efforts would receive greater sympathy and more cordial support than have heretofore been accorded them. Our labors are entirely in behalf of the public, and a remarked by an absolute unselfishness. We are educators and public benefactors in the fullest meaning of the term. Every sick person, rich or poor, profits by the educational benefit his physician derives from attendance on these meetings. Every citizen receives the protection procured through the instrumentality of these societies in the passage of protective statutes and the promulgation of the laws of hygiene.

Every State medical society (except those of Arkansas, Idaho, Indian Territory and New Mexico) publishes its transactions anually either in book form or as a monthly journal. (Minnesota, Mississippi, Montana and hereafter Pennsylvania, in the form of a monthly journal, all the others in an annual volume.) These publications constitute about 22,160 pages. Deducting 2,160 pages for minutes, we see 20,000 pages devoted to scientific or strictly technical matter. Some of the most valuable contributions to medical literature appear in these transactions. It is imposible to estimate their importance. The best of them find their way into medical journals and reach thousands of readers beyond the confines of the State from which they emanate. Every article published in these transactions, and the author of it, is listed in the great monthly publication, *Index Medicus*. This gives authors ready access to special articles which facilitate systematic study of diseases and conditions influenced by widely varying topographical and climatic influences. Thus authors and teachers are enabled to make use of this vast and valuable annual collection of medical and surgical literature, much of which is by these means permanently preserved in text-books and disseminated in the class-room.

A still more important work, if possible, has been accomplished by the State societies in practical legislation, and I regret to record the fact, in spite of the most unreasoning and unreasonable opposition on the part of the people. The passage of laws such as we have advocated before national, State and municipal legislative bodies have invariably been for the protection of our neighbors, and never in any way such as would result in personal or professional gain to us. On the contrary, they have been for the adoption of measures that would inevitably curtail our revenues. Where have you seen men of other professions banded together in leagues, striving to reduce the business from which they gain a livelihood? A medical man as a mere individual can have no more interest in the public weal than any other good citizen; however, his semi-official guardianship of public health impels him to go forward with unselfish purpose to protect those who constitute his charge. I have seen—and so has every other gentleman who has advocated the passage of laws to provide for the protection of the people in the way of dismissing quacks and pretenders and establishing protective health measures—the very ones we sought to benefit rise up in violent opposition to our undertaking, as if our purpose looked to professional and personal aggrandizement instead of bestowing a boon upon our fellows. How dull and blind such are!

Many of us recall the day when no qualifications to practice medicine were called for by the State beyond the mere payment of the license tax. The most ignorant, unscrupulous, debased person who paid this tax to the State was constituted by the law a doctor with all the rights and privileges enjoyed by the learned, upright and beloved physician. Happily this has come to an end, and to-day we see existing in almost every State and Territory laws regulating the practice of medicine—they would be more properly styled laws for the protection of the people. These laws are more or less effective. Some are nearly perfect, others as yet deficient. It is to be hoped that within the fulness of time all will be brought up to the standard of the New York law, which should serve as a model for future statutes and a guide for amendments to present imperfect laws. I append a list of those States which have enacted statutes regulating the practice of medicine,* and I am prepared to assert upon official information that the State Medical Society in every State enjoying such laws, except Oregon and Utah, was the direct instrument and means by which these beneficent measures were secured to the people. These laws had but one object, to furnish the people with

*See table on page 500.

only properly equipped practitioners. They called into existence many State examining boards, whose function was to select qualified men. It was soon made apparent that even many of those holding diplomas were not qualified. Careless and inferior schools saw their graduates excluded from the privileges of practice, and their classes diminished because young men contemplating the study of medicine began to choose their school with discrimination, having in view the purpose of properly fitting themselves to meet the requirements of the various examining boards. The effect of all this was to elevate the standard of medical education. New subjects were added to college curricula, practical work was required, the time of study lengthened, and more rigid examinations exacted. In consequence of these laws we observe an improvement in the material from which medical men are formed; a better preliminary education of medical students, the higher technical education of doctors, the admission into practice of only well-qualified physicians and the rapid disappearance of the pretender and the quack—all of which results to the great benefit of the people.

The creation of legal requirements to enter into the practice of medicine, accomplished by the various State societies acting independently of each other, has made a profound impression on the medical schools all over the country. Without exception, they have advanced their requirements for entrance, prolonged their terms of study, and perfected their methods of teaching. They have also entered into hearty co-operation with the licensing boards and have banded themselves into a league whose purpose it is to still further elevate the standard of education and secure uniform methods of teaching. The natural consequence of these distinct advances and the attraction of public attention to the work in this line of societies and licensing boards brought about the formation of the "Confederation of National Examining and Licensing Boards." This organization is yet young but rapidly pushing forward to accomplish a splendid work, and it deserves the hearty support and co-operation of every State society and licensing board. Its objects are to exalt medical education, to establish a fixed standard of requirements to enter the profession of medicine, to procure uniformity of laws regulating the practice of medicine, and reciprocity between the various State organizations by means of which the license of one board will be recognized and accepted everywhere. These are aims greatly to be desired, and from the present trend of legislation it is not too much to hope that they will be speedily accomplished. There is no need for a National Board of Licensers. All of this should be regulated by the representatives of the various States assembled in the councils of the

National Confederation agreeing upon definite methods and prerequisites.

The laws regulating the practice of medicine may be divided into three classes (C. E. Busey, M. D., Washington, D. C.):

*First Class*, Virginia and others. A diploma confers no right to practice and has no legal value. The right to practice is determined by examinations before boards of examiners enacted by law.

*Second Class*, California and others. The diploma is subject to the supervision of some designated body, vested by law with authority to determine its validity as evidence of its possessor's qualification to the practice of medicine. Failing the possession of such diploma, the right to practice may be acquired by passing a satisfactory examination.

*Third Class*, Where any kind of a diploma from any "chartered" medical institution is sufficient to entitle its holder to registration and the right to practice.

As long as this dissimilarity in statutes regulating the practice of medicine exists, it is easy to see how the States of the second and third class must suffer by becoming the dumping-ground for the refuse and offal rejected by States of the first class. Self-protection alone, it would seem, should induce them to meet the required standard, and thus secure their citizens against the evils and perils of an ignorant medical profession.

The results obtained in another department of legislation are not less striking. In all the States having laws regulating the practice of medicine, except Colorado, Georgia, Idaho, Louisiana, Montana, New Jersey, Oregon, Texas, Utah and West Virginia* State boards of health have been secured to the people by the efforts and through the wisdom of the State medical societies.

In every instance the initiative was taken by the State Society and successfully carried out. These boards have been of inestimable value to the public in the way of promulgating the laws of hygiene, in the prevention of epidemics, the suppression of those already existing, and the conduct of commerce in times of public fear. It is true that some of these boards are not sufficiently supplied with means to properly equip them for efficient work, yet their very existence is a safeguard to the people, and, although deprived of State help, they accomplish much good. State and municipal governments and the people are blind to their own welfare not to give more general encouragement to those guarding their health, wealth and safety.

Some of us remember when epidemics of smallpox, cholera, or yellow fever would devastate entire cities and destroy com-

*These States have boards of health which, however, were not established upon the suggestion or by the aid of the State societies.

merce. Before boards of health were interposed between these destroyers and the people, by the efforts of our State societies, there was scarcely a year that some section of our country was not scourged by one or more of these dread visitors. How this is all changed by the presence in almost every State, city and town of an efficient board of health! These bodies instruct the people in preventive measures; look to the household safety by house-to-house inspection; pass on the efficiency of the sanitary arrangement of contemplated buildings; teach hygiene in public and private schools; institute compulsory vaccination; require the presence of contagious or infectious diseases to be reported, and keep a surveillance on all such cases; isolate them as far as possible; disinfect contaminated buildings; destroy all sources of infection; see to the wholesomeness of our foodstuffs; inspect our meats, fish and vegetables; prevent deleterious adulteration of manufactured foods; purify our markets; cleanse our streets and dispose of offensive garbage; stand guard over our drinking water, and in a thousand other ways look to the safety of the public health. All these measures, executed by men who are either not paid at all, or if compensated, insufficiently so, have added greatly to the average of human life and given to the people a sense of security they scarcely deserve, because they have not borne their part in procuring the means which have brought them these blessings.

These boards, in times of pestilence, stand in the same relation to towns and cities which military garrisons do in times of war. They are ever on the watch, and their vigilance never sleeps. Quarantines are laid on or against stricken cities or communities; possible means of spreading infection or contagion are prohibited; seaports are scrupulously protected; inspection is everywhere required; suspects are isolated; merchandise is dealt with according to safe protective methods, and every precaution taken by these faithful servants to prevent spread of disease. Pestilence is thus checked and life and property saved. It is not difficult to estimate the commercial loss to a city from the presence in it of an epidemic of a contagious or infectious disease. What would happen to one of our seaport towns to-day if she should become the center of an outbreak of yellow fever or cholera? Immediately upon the appearance of infection, a cordon or corral is thrown around her; the ports of the world are closed against her; her incoming freights seek other markets; her exports are arrested; communication with her is prevented; her people are locked within her gates to await their fate; mercantile houses close; banks suspend; values shrink; factories shut down; capital lies idle; her wealth dwindles, and fortunes disappear. Millions of money are forever lost to her, and years are required for the repair of her losses. Other communities suffer as well.

The hindered imports and exports are important to remote communities, and their arrest works disaster to trade in general. When these facts are realized, is it not singular that our philanthropic aims are not more earnestly seconded by the people and State? We stand between them and danger. They rely on us to protect and save them when disaster threatens or is upon them. Why will they not recognize in our attempts safety to property and the enjoyment of peaceful protection, and lend us their moral support and vote public money to the protection of public health?

I am constrained to believe that the derelictions of the people are in large part due to ignorance. It is our duty, then, as members of this Association, in connection with others of a similar character scattered all over the country, to enlighten our constituency by personal instruction. Let us not hide our light under a bushel, but plainly proclaim to the world why we exist, what we have accomplished for the good of our fellows, and ask for a generous and intelligent co-operation in the prosecution of our good work, much of which is yet to be done.

In conclusion, let me make a single recommendation. We have worked with only the purpose of benefiting our fellows. Let us trust that we may be permitted to do this in our own sagacious way. Let our law-makers realize that we alone are competent to choose those who are to execute the function of licensers of practitioners and health officers. We know the fittest men for these high duties; let us be given the selection of them. Remove these appointments entirely from politics and put them where they properly belong—into the hands of the wise men whose wisdom and foresight have secured them to the people as the instruments of this protection.—*Virginia Medical Semi-Monthly.*

